# Associations of water, sanitation, and hygiene with typhoid fever in case–control studies: a systematic review and meta-analysis

**DOI:** 10.1186/s12879-023-08452-0

**Published:** 2023-08-29

**Authors:** Chaelin Kim, Gerard R. Goucher, Birkneh Tilahun Tadesse, Woojoo Lee, Kaja Abbas, Jong-Hoon Kim

**Affiliations:** 1 International Vaccine Institute, Seoul, South Korea; 2 Graduate School of Public Health, Seoul National University, Seoul, South Korea; 3 London School of Hygiene & Tropical Medicine, London, UK

**Keywords:** Typhoid fever; Water, sanitation, and hygiene (WASH), Case-control study, Intervention strategy, Bayesian meta-analysis

## Abstract

**Background:**

Water, sanitation, and hygiene (WASH) play a pivotal role in controlling typhoid fever, as it is primarily transmitted through oral-fecal pathways. Given our constrained resources, staying current with the most recent research is crucial. This ensures we remain informed about practical insights regarding effective typhoid fever control strategies across various WASH components. We conducted a systematic review and meta-analysis of case-control studies to estimate the associations of water, sanitation, and hygiene exposures with typhoid fever.

**Methods:**

We updated the previous review conducted by Brockett et al. We included new findings published between June 2018 and October 2022 in Web of Science, Embase, and PubMed. We used the Risk of Bias in Non-Randomized Studies of Interventions (ROBINS-I) tool for risk of bias (ROB) assessment. We classified WASH exposures according to the classification provided by the WHO/UNICEF Joint Monitoring Programme for Water Supply, Sanitation, and Hygiene (JMP) update in 2015. We conducted the meta-analyses by only including studies that did not have a critical ROB in both Bayesian and frequentist random-effects models.

**Results:**

We identified 8 new studies and analyzed 27 studies in total. Our analyses showed that while the general insights on the protective (or harmful) impact of improved (or unimproved) WASH remain the same, the pooled estimates of OR differed. Pooled estimates of limited hygiene (OR = 2.26, 95% CrI: 1.38 to 3.64), untreated water (OR = 1.96, 95% CrI: 1.28 to 3.27) and surface water (OR = 2.14, 95% CrI: 1.03 to 4.06) showed 3% increase, 18% decrease, and 16% increase, respectively, from the existing estimates. On the other hand, improved WASH reduced the odds of typhoid fever with pooled estimates for improved water source (OR = 0.54, 95% CrI: 0.31 to 1.08), basic hygiene (OR = 0.6, 95% CrI: 0.38 to 0.97) and treated water (OR = 0.54, 95% CrI: 0.36 to 0.8) showing 26% decrease, 15% increase, and 8% decrease, respectively, from the existing estimates.

**Conclusions:**

The updated pooled estimates of ORs for the association of WASH with typhoid fever showed clear changes from the existing estimates. Our study affirms that relatively low-cost WASH strategies such as basic hygiene or water treatment can be an effective tool to provide protection against typhoid fever in addition to other resource-intensive ways to improve WASH.

**Trial registration:**

PROSPERO 2021 CRD42021271881.

**Supplementary Information:**

The online version contains supplementary material available at 10.1186/s12879-023-08452-0.

## Background

Typhoid fever, an infection caused by *Salmonella enterica* serovar Typhi (*S*. Typhi), is a global public health problem. An estimated 11 to 20 million typhoid fever cases including 128,000 to 161,000 deaths occur each year [1–4] with the majority in low- and middle-income countries (LMICs) [5, 6]. Although several effective treatment and prevention strategies are available [7], improving water, sanitation, and hygiene (WASH) is considered key to preventing typhoid fever considering that *S*. Typhi is transmitted via fecally contaminated water or food [8].

Understanding the relative strengths of the association between different components of WASH and typhoid fever may lead to more cost-effective strategies for implementing various WASH components that can provide the strongest protection against typhoid fever [9]. Designing such a strategy requires a detailed understanding of the strength of the association between different components of WASH and typhoid fever.

Population levels of access to improved WASH are monitored by the WHO/UNICEF Joint Monitoring Programme for Water Supply, Sanitation and Hygiene (JMP) in over 190 countries since 1990 [10]. The JMP WASH classification has three categories – drinking water, sanitation, and hygiene – and each category has service ladders indicating different levels of improvement. For instance, the drinking water category has five service ladders: safely managed, basic, limited, improved, unimproved, and surface water. JMP estimates on each of the different categories can be compared across each of the 190 countries that cover almost all of the LMICs.

Understanding the strength of the association between the levels of WASH and typhoid fever risk can create an opportunity to leverage the efforts of the JMP to better understand the risk of typhoid fever within and across countries. Although an association between typhoid fever and the levels of WASH practices is evident, the strength of this association tends to differ across studies. The systematic review and meta-analysis by Mogasale et al. [11] summarized the findings from case–control studies on the association between the levels of WASH and typhoid fever. This study focused only on the drinking water source and exposure categories of the included studies were not classified according to the JMP WASH categories. The systematic review and meta-analysis by Brockett et al. [12] included all three categories of WASH and categorized WASH exposures from case–control studies according to JMP WASH classification, but was applied in a broader level without using specific service ladders. Both studies included findings based on Widal-confirmed typhoid fever cases in addition to cases confirmed through blood culture, which may introduce bias because of the low specificity of the Widal test [13].

In this study, we aim to improve the estimates for the association between WASH exposures and typhoid fever by including new findings published since the previous review done by Brockett et al. [12], applying a rigorous risk of bias assessment, and clarifying the association between the JMP WASH categories and WASH exposures measured in case–control studies. Our study findings will be useful to infer actionable insights on the most effective ways to prevent the spread of typhoid fever and the ways to leverage the WHO/UNICEF JMP WASH data to explore the potential burden of typhoid fever.

## Methods

### Search strategy

We searched three databases – Web of Science, Embase, and PubMed – to find peer-reviewed articles in English. In each database, we searched using the following search terms: (“case control” OR “case–control”) AND “typhoid”. The search terms were consistent with the previous review done by Brokett et *al.* [12] except that we did not include “retrospective” to restrict our search to case–control studies. We restricted our search to articles published from June 2018 through Oct 2022 to identify articles that were published after the publication of Brockett et al. study [12], which included articles published between January 1990 and June 2018.

### Inclusion and exclusion criteria

We developed inclusion and exclusion criteria based on the population, intervention, comparison, outcomes, and study design (PICOS) framework [14]. These predefined criteria were included in the protocol published in PROSPERO [15]. Eligible study populations encompassed populations of all ages, genders, and socioeconomic statuses living in low- and middle-income countries as defined by the World Bank [16]. Studies would be eligible for inclusion if they considered one of five WASH exposure categories, specifically: water source, water management, water treatment, sanitation, and hygiene. We excluded studies that were meant to evaluate vaccine efficacy in which the nature of interactions between WASH exposures and vaccination was not clear. Studies were considered eligible if they investigated association between typhoid fever and at least one WASH exposure using an odds ratio (OR).

### WASH exposure categories

Studies varied in their WASH exposures, and we tried to systematically map the WASH exposures from included studies to the JMP WASH categories and service ladders (Table 1). The JMP provided service ladders for each of the three WASH categories: drinking water, sanitation, and hygiene. In addition to these three categories, we used two additional categories of water treatment and water management to delve into other important characteristics of water exposures. These two categories were also used in the previous review by Brockett et al*.* [12]. However, for hygiene, we aimed to utilize the JMP service ladder, which specifically focuses on handwashing practices by assessing the availability of handwashing facilities with soap and water at home. While we acknowledge the substantial role of food hygiene in typhoid infection, we did not include it in our study as we chose to follow the JMP's definition of hygiene [17].

**Table 1:** WASH-related exposures from included studies and corresponding WASH service ladders. The following table includes the WASH category, service ladders and examples of WASH exposures. This categorization was used to classify the extracted data for the meta-analysis

WASH category	Service ladders	Example WASH exposures from included studies
Water source††	Improved (Safely managed, Basic, Limited)*	Drinking piped water only, tube well water, etc
	Unimproved	NA
	Surface water	Drinking river water, surface water, stream water, unboiled surface water, etc
Sanitation	Safely managed	NA
	Basic	NA
	Limited	NA
	Unimproved	Unimproved pit latrine
	Open defecation	Places used to defecate (field, pond, river, canal, nearby stream), sewage disposal directly to the environment etc
Hygiene	Basic	Use of soap for handwashing, soap hand wash before food/after defecation/after urination, soap available to wash hands, soap observed in home
	Limited	Soap not available near toilet, no use of soap for handwashing
	No facility	NA
Water treatment†	Treated water	Drinking purified water, boiled water in home, treated water in home, disinfected water at home using boiling or filtration
	Untreated water	Drinking untreated water, drinking unboiled water, grossly contaminated home water
Water management†	Safe water storage	Storage of water in covered container, narrow-mouth container, metal covering, wide mouthed container with lid, a narrow mouthed container (with lid)
	Unsafe water storage	Dirty container for storing drinking water

^*^ While JMP WASH classification provides five service ladders, WASH exposures from individual studies do not provide sufficient descriptions to match any of the three service ladders. We grouped the three ladders into a single category called improved water source. The definition of “improved” is also defined by the JMP [10]

^†^ These categories are not part of the JMP WASH classification

^††^ The original category name is “drinking water” under the JMP classification

*NA* Not available

We checked weather specific WASH exposures from included studies matched the JMP ladder definitions. If they matched one of these definitions, the exposure would be placed into the corresponding JMP ladder. For instance, basic in the JMP hygiene ladder was defined as “availability of a handwashing facility with soap and water at home”. Accordingly, we classified relevant exposures such as the use of soap for handwashing or soap available to wash hands under the basic hygiene category. We used the five WASH categories with 15 subcategories to synthesize the findings on the association between the WASH characteristics and typhoid fever.

### Data extraction

We had three reviewers (CK, GG, JHK). Two reviewers assigned to each study determined the eligibility of articles in two separate phases. Any disagreements were resolved by discussion. Initially, titles and abstracts were screened to ensure that the studies used the case–control methodology, that the outcomes are typhoid cases, and that the context was in LMIC. Then, full manuscripts were read to ensure that articles met all of our PICOS criteria. Two reviewers (CK, GG) extracted data from the included studies, including author information, publication year, case/control definitions, WASH exposures, diagnostic methods, country, and effect size (odds ratio) for individual exposures. Google Sheets was used to manage the data.

### Risk of bias assessment

We assessed the risk of bias of the included studies using the Cochrane Risk of Bias in Non-Randomized Studies of Interventions (ROBINS-I) tool [18] in seven domains: 1) confounding, 2) selection, 3) intervention classification, 4) intervention deviation, 5) missing data, 6) outcome measurement, and 7) selective reporting. Based on the assessment results in each domain, the studies were labeled as having a low, moderate, serious, or critical risk of bias. Two authors (CK, JHK) examined the risk of bias independently, and any discrepancies were resolved by discussion.

### Statistical analysis

Data from studies that did not have critical risk of bias were used to generate the pooled estimates. Studies that did not use culture-confirmed cases were excluded in any data synthesis. The analyses were performed using the R statistical software (version 4.1.3). We developed a series of Bayesian random effects models using the *brms* package [19] to estimate the pooled ORs with 95% credible intervals (CrIs) for each exposure category with more than two studies. Random effects models were utilized as we assume that true effects may vary for each study depending on the contexts. Bayesian meta-analyses are particularly useful when the number of studies is small and enable us to use prior knowledge [20]. We assessed the possibility of publication bias through visual inspection of the funnel plots (Appendix B). The repository for the data and software code of this study are publicly accessible at the GitHub repository [21].

## Results

### Overview of included studies

**Fig. 1:**
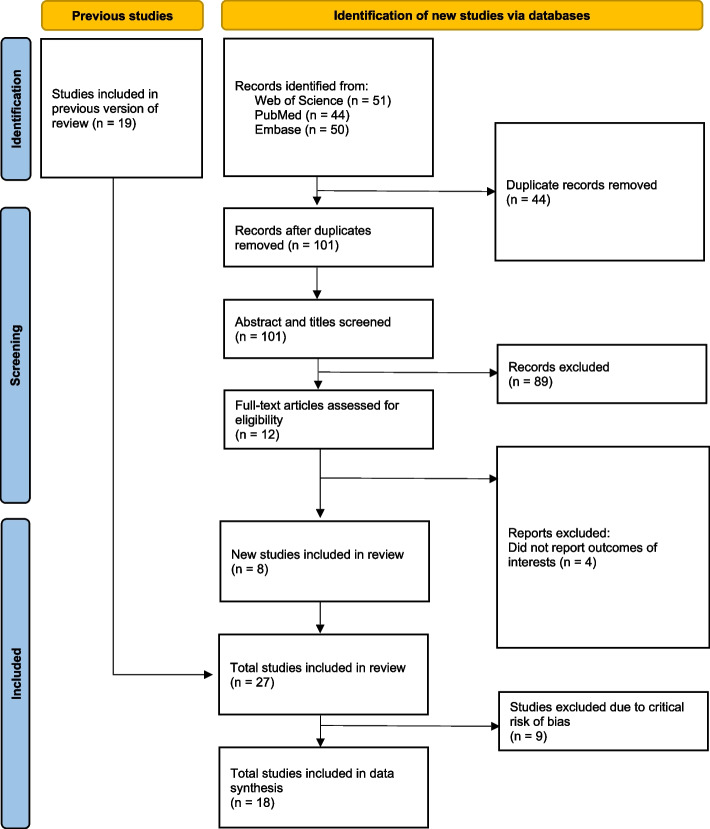
PRISMA flow diagram. The PRISMA (Preferred Reporting Items for Systematic Reviews and Meta-Analyses) flow diagram shows the number of articles at the different phases of identification, screening, and inclusion in the systematic review and meta-analysis

The PRISMA flow diagram (Fig. 1) depicts the different phases of a systematic review. We identified 51, 44, and 50 articles from Web of Science, PubMed, and Embase, respectively. We obtained 101 unique articles after removing the duplicates. After reviewing the title and abstract, we excluded 89 non-eligible articles and reviewed the full-text copies of 12 studies. Following the full-text review, eight new studies were included in our review in addition to the 19 studies included in the previous review conducted by Brockett et al. [12], hence making a total of 27 studies included in our review. All extracted data from the included studies can be found in Appendix A. The newly identified studies are from the Democratic Republic of Congo, Fiji, India, Malawi, Pakistan and Uganda [22–29]. Among the 27 included studies, 18 studies (67%) used blood culture to define cases. The included studies showed variability in terms of the WASH exposures studied and the variables controlled when estimating the association between these WASH exposures and the odds of typhoid fever (Table 2). After removing the studies with potentially critical risk of bias, we included 18 studies for meta-analyses.

**Table 2:** Characteristics of studies. Characteristics of included studies in the systematic review with the JMP WASH category, country, exposures, effect size (odds ratio or adjusted odds ratio), and diagnostic methods

**Study**	**Country**	**JMP WASH Category**	**Exposures**	**Effect Size** **(Odds Ratio)**	**Diagnostic Methods**	**Controlled Variable**††
Alba et al. 2016 [30]	Indonesia	Water Treatment (Untreated water)	Water treatment before drinking (Never)	1.68 (0.99–2.82)†	Serology and culture	
		Sanitation (Open defecation)	Places used to defecate (Field)	0.98 (0.72–1.34)		
		Sanitation (Open defecation)	Places used to defecate (Pond/river/canal)	0.77 (0.56–1.06)†		
		Hygiene (Limited)	Soap near toilet (Never)	4.4 (2.0–9.65)†		
Aye et al. 2004 [31]	Myanmar	Water Source (Surface water)	Drink untreated river water	12.5 (2.8–75.3)*	Diazo urine test or positive widal test	Drink untreated river water, Contact with typhoid patient, Hand washing with soap, Travel to other place
		Hygiene (Basic)	Hand washing with soap	0.15 (0.03–0.81)*		
Batool et al. 2022 [27]	Pakistan	Water Treatment (Untreated water)	Method of water purification used (None)	0.93 (0.56–1.56)†	Blood culture	
Bhan et al. 2002 [32]	India	Water Management (Unsafe water storage)	Dirty container for storing drinking water	1.99 (0.6–6.65)*	Blood culture	Ownership of dwelling, Nuclear family, No family member literate, Typhoid case in the family, Dirty container for storing drinking water, Nonuse of soap for washing hands, Water or drinks outside home, Lunch or dinner outside home, Consumption of ice cream
	India	Hygiene (Limited)	Nonuse of soap for washing hands	1.82 (1.04–3.21)*†	Blood culture	
Bhunia et al. 2009 [8]	India	Water Source (Improved)	Drinking water (Piped water only)	7.3 (2.5–21)	Widal test	
		Water Source (Improved)	Drinking water (Tube well water)	0.25 (0.08–0.75)		
		Water Treatment (Treated water)	Drinking water (Purification)	0.44 (0.19–1.0)		
		Water Management (Safe water storage)	Drinking water (Covered container)	0.25 (0.08–0.75)		
		Water Management (Safe water storage)	Drinking water (Narrow mouth container)	0.35 (0.15–0.76)		
		Hygiene (Basic)	Soap hand wash before food	1.9 (0.8–4.4)		
		Hygiene (Basic)	Soap hand wash after defecation	0.3 (0.12–0.75)		
		Hygiene (Basic)	Soap hand wash after urination	0.08 (0.03–0.26)		
Brainard et al. 2018 [22]	Democratic Republic of Congo	Hygiene (Not classified)	Wash hands after defecating (Always)	2.71 (1.4–5.28)*	Diagnosis of clinical signs and Blood/bone marrow culture/duodenal fluid culture	Plate sharing, Occupation of head of household, Tap water is ever used, Wash hands after defecating, Water source chosen because it is protected, Visible urine/faeces at latrine
Bruh et al. 2017 [33]	Indonesia	Hygiene (Not classified)	Poor hand washing practice before eating	4.295 (1.232–14.969)	TF Tubex	
Gauld et al. 2019 [23]	Malawi	Hygiene (Basic)	Soap available to wash hands after toilet use in the previous 3 wk	0.6 (0.4-0.98)*†	Blood culture	Seeking care at QECH if child is severely ill, ≥ 1 household members admitted to hospital for febrile illness and cleaning with river water, > 1 drinking water source used, Child spends the day at school, preschool, nursery, or any other daycare, Cooking and cleaning using water from an open dug well, Family grows crops, Age, Distance from household to primary water source, No. of days water is stored, Experienced water shortage in the house or surrounding area, Soap available to wash hands after toilet use, Stores drinking water in drum, Used stream or river water for drinking
Giri et al. 2021 [28]	India	Water Treatment (Treated water)	Treatment of household water (boiling, filtration, reverse osmosis)	0.45 (0.25–0.80)*†	Blood culture	Treatment of household water (boiling, filtration, reverse osmosis), Washed produce before eating, Consumption of street food by mother during past week
Kabwama et al. 2017 [34]	Uganda	Water Source (Not classified)	Consumed implicated drinks	1.9 (0.68–5.10)	Diagnosis of clinical signs	
Karkey et al. 2013 [35]	Nepal	Water Management (Safe water storage)	Metal covering of water storage	0.22 (0.1–0.6)	Blood culture	
Luby et al. 1998 [36]	Pakistan	Water Treatment (Treated water)	Home water (Clean home drinking water)	0.9 (0.4–1.9)†	Blood culture	
		Water Treatment (Untreated water)	Home water (Grossly contaminated)	1.1 (0.6–2.1)†		
Luxemburger et al. 2001 [37]	Vietnam	Water Source (Surface water)	Drinking river water	1.8 (0.8–5.6)†	Blood culture	
		Water Treatment (Untreated water)	Drinking unboiled water	1.5 (0.8–3.3)†		
		Sanitation (Open defecation)	Defecation in a fish pond or river	1.1 (0.5–3.2)†		
Mermin et al. 1999 [38]	Tajikistan	Water Treatment (Treated water)	Boiled water in home	0.2 (0.05–0.6)*†	Blood and stool culture	Drinking unboiled water (or boiling water in the home), Obtaining water from an outside tap, Eating butter, Eating apples
		Water Treatment (Untreated water)	Drinking unboiled water	9.6 (3.0–34.0)*†		
Mirembe et al. 2019 [26]	Uganda	Water Treatment (Treated water)	Do you treat your drinking water?	9.23 (4.84–17.61)*	Diagnosis of clinical signs	Level of formal education, water treatment for the drinking water, washing hands with soap
		Hygiene (Basic)	Do you wash hands with soap?	1.81 (0.95–3.45)*		
Muti et al. 2014 [39]	Zimbabwe	Water Treatment (Treated water)	Boil drinking water	0.24 (0.07–0.90)*	Diagnosis of clinical signs	Water from well, Burst sewer pipe within 500 m of home, Typhoid contact at home, Store water in wide mouthed container with lid, Boil drinking water, Storage of water in a narrow mouthed container with lid
		Water Management (Safe water storage)	Store water in wide mouthed container with lid	3.68 (1.62–8.35)*		
		Water Management (Safe water storage)	Storage of water in a narrow mouthed container with lid	0.43 (0.22–0.8)*		
Nyamusore et al. 2018 [40]	Rwanda	Hygiene (Not classified)	Washing hands after using the latrine (Sometimes or never)	1.78 (1.21–2.62)*	Diagnosis of clinical signs	Time spent in the camp, Level of completed education, Family members treated for typhoid fever in the past 3 months, Heard about typhoid fever before the outbreak, Washing hands after using the latrine, Most common source for food, Frequency of jerry-can washing
Prasad et al. 2018 [24]	Fiji	Water Source (Surface water)	Drank from an alternative water source (surface water source)	3.61 (1.44–9.06)*†	Blood culture	Drank from an alternate water source (surface water source), Water not always available from main source, Did not wash produce before eating, Had any unimproved sewerage/damaged improved sewerage system, Undamaged, improved, municipal sewerage, Unimproved pit latrine, No toilet/Open defecation, Damaged improved, municipal sewerage, Improved pit latrine, Intact septic, High handwashing frequency after defecation, Use soap for handwashing
		Water Source (Surface water)	Main household water source (Surface water source)	1.28 (0.35–4.70)		
		Water Treatment (Treated water)	Treated water in house	0.89 (0.57–1.39)†		
		Water Treatment (Untreated water)	Drank untreated water	1.8 (1.07–3.03)†		
		Sanitation (Unimproved)	Unimproved pit latrine	49.47 (9.42–259.92)*		
		Sanitation (Open defecation)	No toilet (Open defecation)	9.87 (0.85–114.35)*†		
		Hygiene (Basic)	Use soap for handwashing	0.61 (0.37–0.95)*†		
Qamar et al. 2018 [25]	Pakistan	Water Source (Not classified)	Unsafe drinking water	1.19 (0.8–1.78)*	Blood culture	Unsafe drinking water, Construction material of house, Number of people per toilet in household, Antibiotic use in 4 weeks before illness, History of contact with person with typhoid, Male sex, Eating outside of house
Ram et al. 2007 [41]	Bangladesh	Water Source (Improved)	Pipe to municipal supply	0.5 (0.2–1.1)†	Blood culture	Unboiled water, Foul-smelling water, Use of latrine, Papaya
		Water Treatment (Treated water)	Disinfected water at home using boiling or filtration	0.7 (0.4–1.6)†		
		Water Treatment (Untreated water)	Drinking unboiled water at home	12.1 (2.2–65.6)*†		
		Hygiene (Basic)	Soap observed in home	0.5 (0.2–1.3)†		
Sharma et al. 2009 [42]	India	Water Source (Improved)	Drinking water (Piped water supply at home)	0.4 (0.2–0.9)	Widal test	
		Water Source (Surface water)	Drinking water (Stream water at home)	1.6 (0.9–2.6)		
		Water Treatment (Treated water)	Drinking water (Drinking boiled water)	1.3 (0.6–2.6)		
		Water Management (Safe water storage)	Storage of water (Narrow-mouthed container)	0.4 (0.2–0.7)		
		Sanitation (Open defecation)	Toilet facilities (Nearby stream)	1.5 (0.9–2.7)		
Siddiqui et al. 2008 [43]	Pakistan	Hygiene (Limited)	Soap available near hand washing facility (No)	2.6 (1.1–6.3)*†	Blood culture or serology test	Number of person in household, Soap available near hand washing facility, Use medicated soap, Aware of contact with known typhoid case
		Hygiene (Not classified)	Wash hands after using toilet (Never)	4 (1.6–10.2)		
		Hygiene (Not classified)	Wash hands before meal regularly (No)	1.6 (0.9–2.9)		
Srikantiah et al. 2007 [44]	Uzbekistan	Water Source (Improved)	Drinking water habits outside home (Piped water)	0.6 (0.3–1.6)†	Blood culture	Drinking unboiled surface water outside home, Student as primary occupation, Antimicrobials in 2 weeks before illness onset, Routinely washing vegetables, Dining at a tea-house
		Water Source (Surface water)	Surface water at home	1.9 (0.7–4.9)†		
		Water Treatment (Treated water)	Drinking water habits at home (Boiled water)	0.4 (0.2–0.8)†		
		Water Treatment (Untreated water)	Drinking water habits at home (Unboiled water)	2.1 (1–4.4)†		
		Water Management (Safe water storage)	Storing water (Keep water container covered)	0.2 (0.04–1.1)		
Tran et al. 2005 [45]	Vietnam	Water Treatment (Untreated water)	Drinking untreated water	3.9 (2.0–7.5)*†	Blood or stool culture	Uneducated people, Contact with a typhoid case, Drinking untreated water, Eating shellfish
		Sanitation (Open defecation)	Sewage disposal directly to the environment	2.4 (1–5.8)†		
Velema et al. 1997 [46]	Indonesia	Hygiene (Limited)	Does not use soap when washing hands	29.8 (2.19–407.0)*	Diagnosis of clinical signs and Widal test	University education, Single, Warung, Soap, Wash clothes, Age, Sex
Vighio et al. 2021 [29]	Pakistan	Water Treatment (Treated water)	Boiling drinking water (Yes)	0.3 (0.2–0.7)†	Culture	
Vollaard et al. 2004 [47]	Indonesia	Water Source (Improved)	Drinking water (Piped water)	0.44 (0.19–1.01)†	Blood culture	No use of soap for handwashing, Sharing food from same plate, No toilet in household, Recent typhoid in household, Young age, Flooding, Use of iced drinks, Use of ice cubes, Crowding
		Hygiene (Limited)	No use of soap for handwashing	1.91 (1.06–3.46)*†		

^*^ adjusted odds ratio from multivariate analysis

^†^ estimates included in the meta-analysis

^††^ Controlled variables in the multivariate analysis

### Risk of bias assessment

**Fig. 2:**
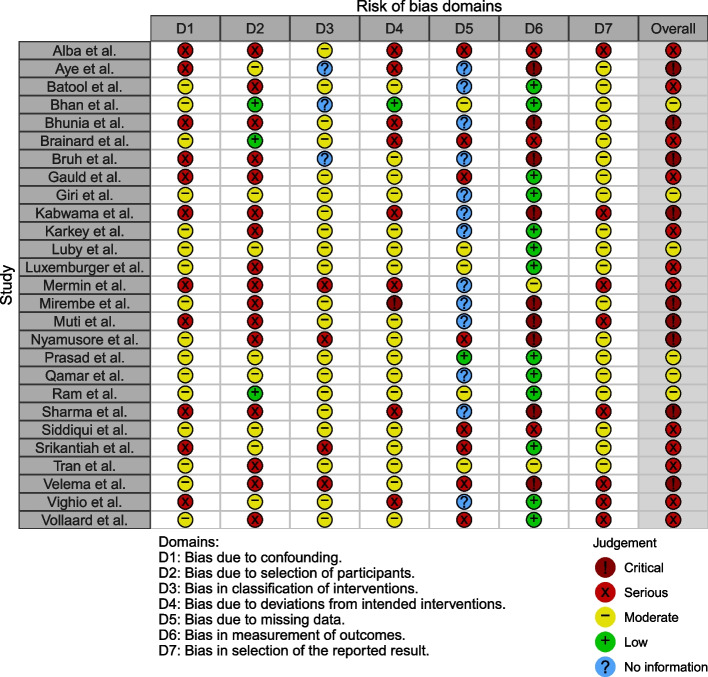
Risk of bias assessment using the Cochrane ROBINS-I tool. The studies included in the systematic review were assessed for risk of bias due to 1) confounding, 2) selection, 3) intervention classification, 4) intervention deviation, 5) missing data, 6) outcome measurement, and 7) selective reporting

Except for six studies, which were categorized as having an overall moderate risk of bias, all other studies were classified as having an overall serious or critical risk of bias (Fig. 2). For the domain of confounding, 16 studies controlled for suspected confounding factors (i.e., age, sex, and socioeconomic characteristics) and were assessed as having a moderate risk of bias even though some level of confounding may still exist because of the inherent nature of the case–control study. For the domains of intervention classification, deviations from intended interventions, and the selection of the reported result, 23, 18, and 19 studies, respectively, were classified as having a moderate or low risk of bias. In addition, 13 studies were labeled as having a low risk of bias as they utilized a culture-confirmed typhoid fever diagnosis. However, 16 studies were rated as having a serious risk of bias as the case–control research design is prone to selection bias. Lastly, 13 studies did not provide adequate information to assess bias due to missing data. The figure on risk of bias assessment results broken down for each risk of bias criterion can be found in Appendix C.

### Meta-analyses

We performed meta-analyses for the seven categories for which there were more than two studies. Overall, the potential risk factors doubled the odds of typhoid (OR = 1.91, 95% CrI: 1.38 to 2.79), while the potential protective factors reduced the odds by half (OR = 0.51, 95% CrI: 0.38 to 0.65) (Appendix E).

### Water source

JMP definition of improved water source includes piped water, protected dug wells, tube wells, protected springs, rainwater, and packaged water. While the improved water source can be further divided using the service ladders (i.e., safely managed, basic, or limited), we used only one category of improved water source because the number of studies is small and descriptions about the exposure were not detailed enough for further classification. Three studies reported data on the improved water source [41, 44, 47]. The pooled estimate of the ORs of improved water source was 0.54 (95% CrI: 0.31 to 1.08) with the between-study heterogeneity (τ) of 0.29.

**Fig. 3:**
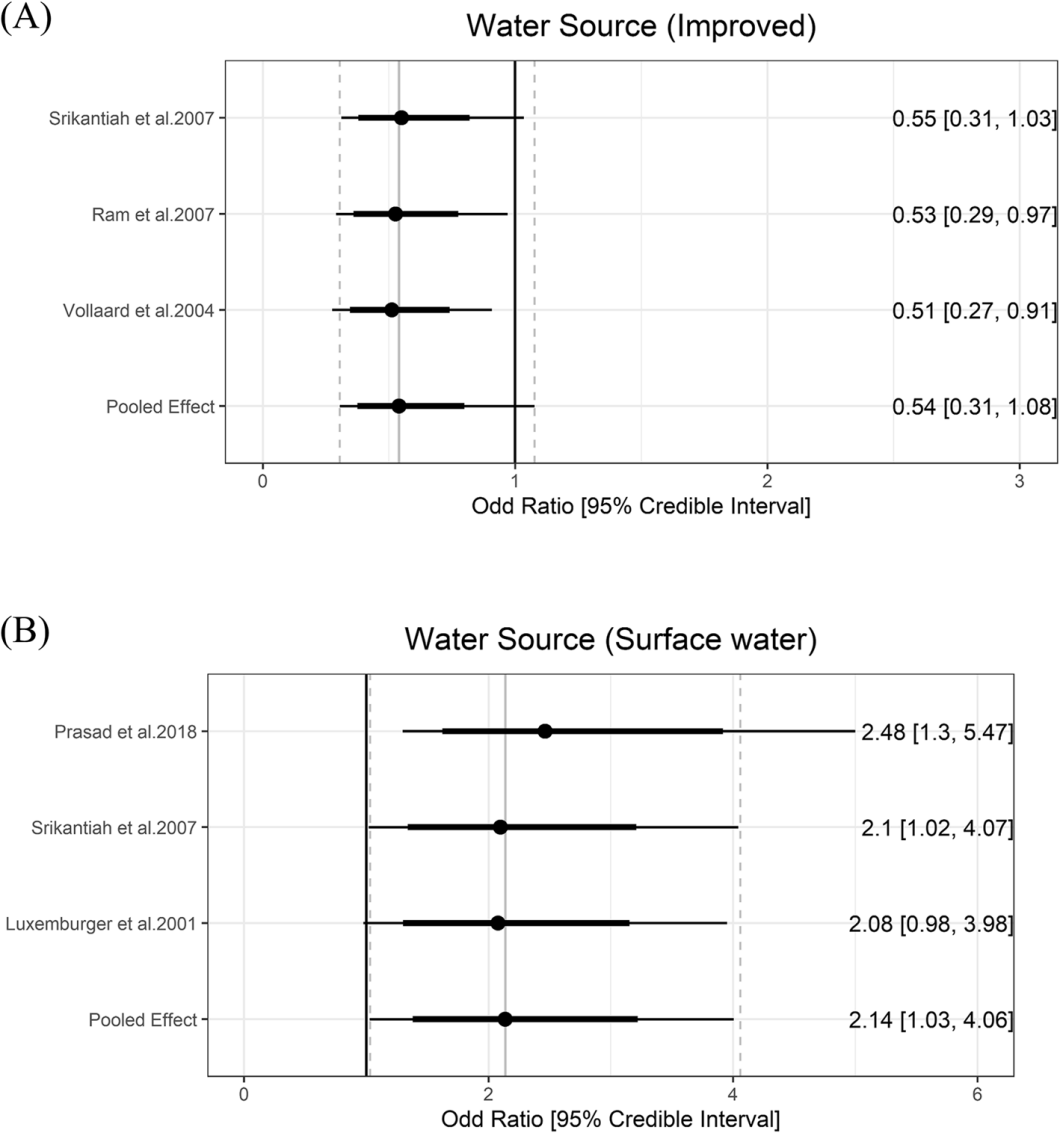
Association between water source and typhoid fever. The forest plot illustrates the association between water source and typhoid fever. Filled circles are posterior median values. Thick and thin black lines show 80% and 95% credible intervals, respectively

Drinking water from an unimproved water source (i.e., unprotected dug well or spring) or directly from surface water are risk factors for typhoid fever. Five values fitted into the surface water group. Surface water sources increased the odds of typhoid by 2.14 (95% Crl: 1.03 to 4.06) with the between-study heterogeneity (τ) of 0.35 (Fig. 3).

### Water treatment

**Fig. 4:**
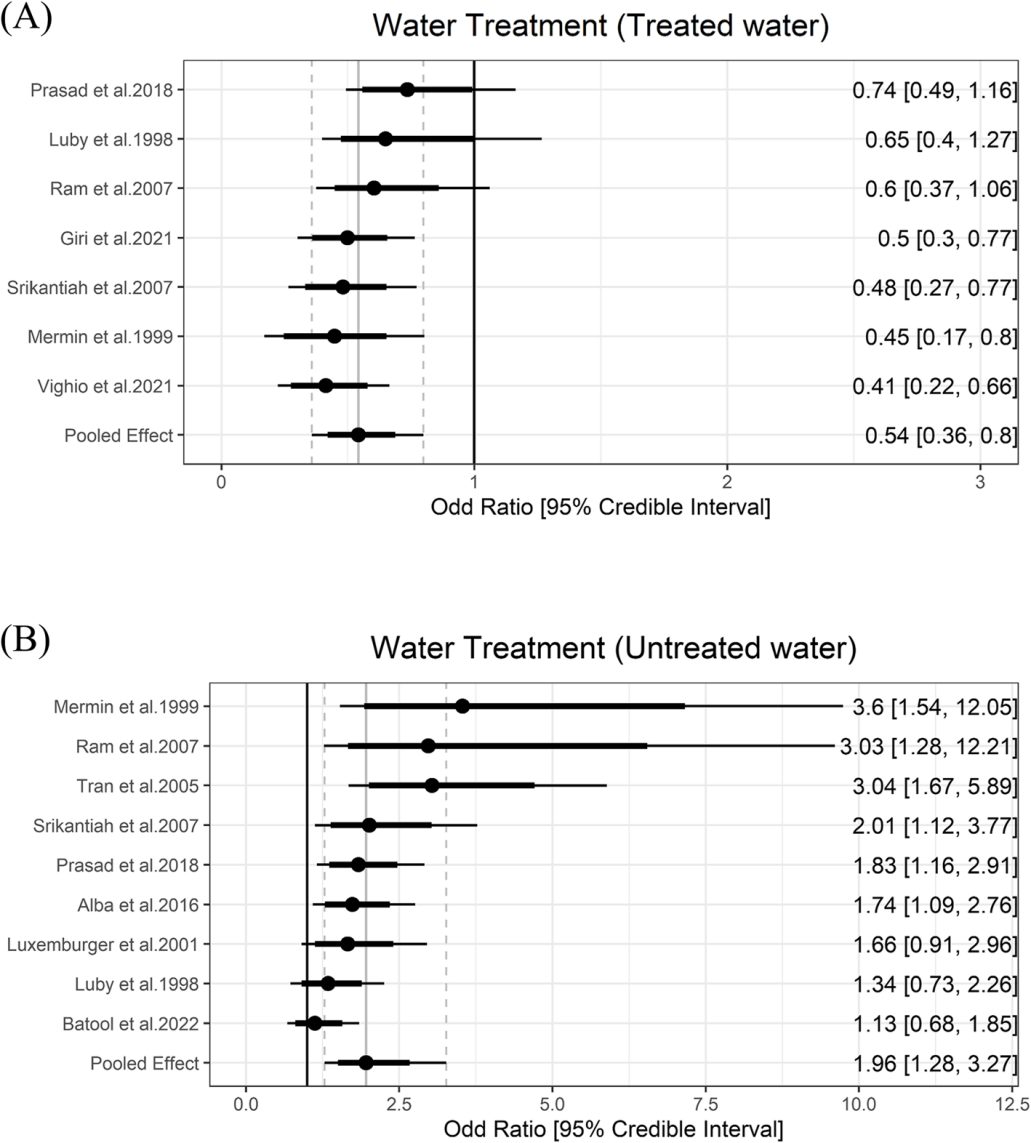
Association between water treatment and typhoid fever. The forest plot illustrates the association between water treatment and typhoid fever. Filled circles are posterior median values. Thick and thin black lines show 80% and 95% credible intervals, respectively

Household water treatment of any kind was included as a predicted protective factor due to prior evidence on decreasing typhoid fever burden [48]. Five studies reported information on water treatment and six exposures were classified as the water treatment group. The meta-analysis showed that any kind of household water treatment lowered the odds of typhoid by 0.54 (95% Crl = 0.36 to 0.8) with the between-study heterogeneity (τ) of 0.37. Using untreated water was a risk factor and increased the odds of typhoid fever by 1.96 (95% Crl = 1.28 to 3.27) with the between-study heterogeneity (τ) of 0.55 (Fig. 4).

### Water management

Safely managed water refers to water being stored in a narrow-mouthed, closed lid to prevent contamination [49], and is considered a protective factor against water-borne diseases. In order to expand the concept of safe water management and get a broader pool of data, we considered narrow-mouthed and/or closed lids in our exposure categories. Two studies measured the association between safely managed water and typhoid fever [35, 44]. Using metal coverage of water storage and keeping water containers covered were associated with around 80% lower odds of having typhoid fever (odds ratio [OR]: 0.22, 95% confidence interval [95% CI]: 0.1 to 0.6; OR: 0.2, 95% CI: 0.04 to 1.1) [3, 4]. Unsafe water management, such as the use of contaminated water storage, is a risk factor, and using dirty containers to store drinking water was associated with double the odds of having typhoid fever (aOR: 1.99, 95% CI: 0.6 to 6.65) [32]. Meta-analysis was not performed in the water management category due to less than three studies.

### Sanitation

**Fig. 5:**
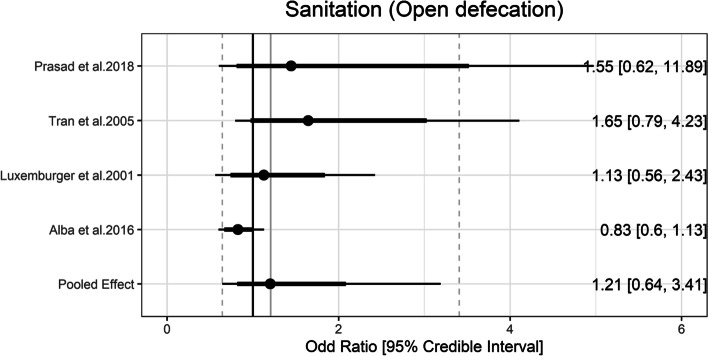
Association between sanitation and typhoid fever. The forest plot illustrates the association between sanitation and typhoid fever. Filled circles are posterior median values. Thick and thin black lines show 80% and 95% credible intervals, respectively

JMP defines improved sanitation facilities as those that prevent human contact with excreta. The categories of improved sanitation facilities can be further divided into safely managed, basic, and limited categories. No exposure categories from studies could be classified into these ladder rungs. Prasad et al. [24] measured that people who were using unimproved pit latrine had nearly 50 times greater odds of having typhoid than the controls (aOR: 49.47, 95% CI: 9.42 to 259.92). On the other hand, the pooled estimate of the ORs of open defecation was 1.21 (95% Crl = 0.64 to 3.41) with the between-study heterogeneity (τ) of 0.56 (Fig. 5).

### Hygiene

**Fig. 6:**
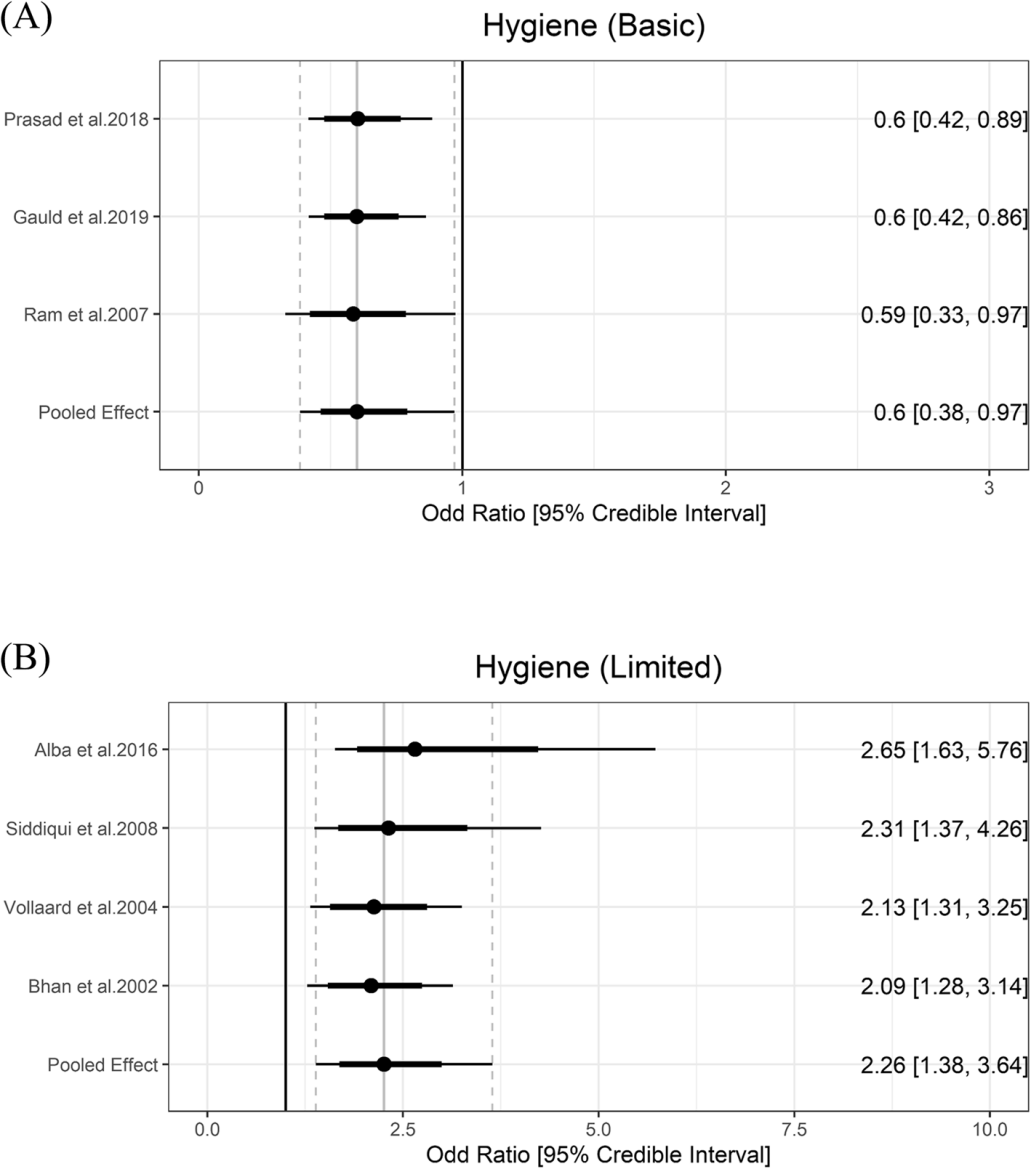
Association between hygiene and typhoid fever. The forest plot illustrates the association between hygiene and typhoid fever. Filled circles are posterior median values. Thick and thin black lines show 80% and 95% credible intervals, respectively

According to the JMP definitions, basic hygiene means that a handwashing facility with soap and water is available at home, and washing hands with soap is protective against diarrhea [48]. In meta-analysis, basic hygiene was associated with lower odds of typhoid (OR = 0.60, 95% Crl = 0.38 to 0.97) with the between-study heterogeneity (τ) of 0.24. Limited hygiene means that a handwashing facility is available at home without soap and/or water. Limited hygiene was associated higher odds of typhoid (OR = 2.26, 95% Crl = 1.38 to 3.64) with the between-study heterogeneity (τ) of 0.29 (Fig. 6).

## Discussion

We conducted a systematic review and meta-analysis of case–control studies to infer the association between water, sanitation, and hygiene (WASH) and culture-confirmed typhoid fever. Our analyses updated the previous estimates of Brockett et al. [12] by adding the data published between June 2018 and Oct 2022 in addition to those included in the previous review and conducting a more comprehensive risk of bias assessment using the ROBINS-I tool. Our pooled estimates for ORs clearly varied from existing estimates while our study confirmed that improved WASH such as treated water and basic hygiene provided substantial protection against typhoid fever and limited hygiene, using untreated water and surface water increased the odds of typhoid fever.

Our meta-analyses of the newly compiled data yielded varied quantitative inferences regarding the association between WASH and typhoid fever compared to prior meta-analyses [12] (Appendix F), particularly in terms of pooled estimates and confidence (and credible) intervals. In terms of protective factors, improved water sources and treated water demonstrated a greater reduction in the odds of typhoid fever than previously reported, while the confidence (and credible) intervals of the new analyses encompassed the estimates from the prior analyses. On the other hand, surface water and limited hygiene were found to increase the odds of typhoid fever to a larger extent and untreated water had a smaller effect on increasing the odds of typhoid fever compared to the prior analyses [12]. This discrepancy could be attributed to variations in the included studies for conducting meta-analyses.

The details of the methods differed between our study and the previous study by Brockett et al. [12], which led to a different data set and consequently different pooled estimates for ORs. Firstly, for the risk of bias assessment, the previous study used the adapted version of the Quality Assessment Tool for Quantitative Studies [50]. On the other hand, we used the ROBINS-I tool and removed studies classified having “critical” risk of bias, which resulted in a smaller number of studies in the meta-analysis. Compared with other risk of bias assessment tools, the ROBINS-I is more systematic and comprehensive and was specifically designed to address weaknesses in other tools [18]. Secondly, We adopted the Bayesian framework as our primary analysis because it could better characterize the uncertainty of the estimates, particularly when the number of studies is small [20], and the difference between these two approaches are most noticeable in the width of confidence or credible intervals. (Appendix F). Thirdly, the previous review [12] included studies in which typhoid fever was confirmed through the Widal test or clinical signs as well as blood culture whereas we included only studies in which typhoid fever was confirmed through blood culture. Clinical symptoms of typhoid fever are not specific enough to differentiate from other enteric diseases [51]. Also, previous literature indicated that Widal test had low sensitivity and specificity (< 80%) and did not recommend using Widal test alone when diagnosing typhoid fever [13]. Fourthly, the previous study included more than one estimate from each sample whereas we only included only one estimate from each sample to avoid violating the assumption of independent findings (i.e., unit-of-analysis error) [52]. For instance, the previous review included two estimates from Alba et al. [30], sometimes treating water before drinking (i.e., sometimes vs. always) and never treating water before drinking (i.e., never vs. always), as inputs for meta-analysis of the untreated water category. We only included one of the two estimates as the two estimates came from the same sample, and we chose the “never vs. always” exposure as we believed it better reflected the risk of untreated water. Similarly, the previous review included both crude and adjusted estimates of the same exposure from the same sample. On the other hand, we included only adjusted estimates in the meta-analysis. Also, when there are multiple exposure estimates from the same study that can be classified into the same JMP WASH category (e.g., use of soap and soap near the toilet can be classified into the hygiene category), the previous review included them in the meta-analysis together. We included only one from each study that fits the JMP definition better (i.e., soap near the toilet in this case) in the analyses. Fifthly, we utilized more detailed WASH subcategories. For instance, although the exposures, ‘washing hands before meals regularly or after using the toilet’, was included in the lack of hygiene category in the previous review, we did not include in our JMP hygiene categories as washing hands does not imply washing hands with soap, which better reflects the JMP hygiene category [43].

Our study has limitations. First, case–control studies included in our meta-analyses varied not only in terms of study place and time, but also in how potential biases were controlled. Therefore, the variances observed in the data set may overrepresent the actual variance of the association between the WASH and typhoid fever. However, the heterogeneities of the OR estimates did not appear to be very high (Appendix F). Second, there were discrepancies across studies in how the WASH exposure data was collected even if they were included in the same JMP WASH category. Only few studies collected data through the direct observation (e.g., observation of soap availability) [32, 41, 43], while the majority of other studies relied on self-reporting, which is prone to recall bias. Third, various WASH indicators may be related to the habits of an individual and thus correlated with one another. This implies that some of the included studies that do not control for other WASH factors can not differentiate the impacts of different WASH components. Some studies controlled for other WASH factors [22–26, 30–33, 36–38, 40, 44], but we did not conduct separate analyses of these due to the small number of estimates available. While the estimates do not seem to vary much between the studies that account for other WASH factors and those that do not, future studies need to pay attention to the multicollinearity among the WASH variables. Fourth, while we used our best judgment to categorize the WASH exposures in case–control studies according to JMP categories, actual WASH exposures included in the same JMP WASH category still varied. Lastly, we only included findings from case–control studies as we were updating the previous review of case–control studies and also the majority of the data are available in the form of case–control studies. Findings from randomized controlled trials [53, 54] and cohort studies [55] are consistent with our analyses. For example, in the clinical trial conducted in Kolkata, India, living in a better WASH environment led to 57% (95% CI: 15—78) reduction in typhoid risk [53].

There is room for future research in this area. While we classified the effect measures (odds ratio estimates) for the WASH exposures on typhoid fever from each study using the updated WASH ladder metric, we had to resort to the old JMP metric of "improved/unimproved" when conducting meta-analyses because of the small number of studies to analyze. In particular, few or no existing studies examined the association between typhoid fever and WASH exposures that can be classified as unimproved water source, safely managed sanitation, basic sanitation, limited sanitation, or no hygiene facility. Future research should further investigate the association between WASH and typhoid fever in this area once more when OR estimates become available. Our findings, when combined with population-level JMP WASH trends, may be used to understand and forecast the population-level risk of typhoid fever, which can provide essential insights for decision-makers. Since the population levels of WASH have been monitored since 1990 in 191 countries, one can also analyse the longitudinal data to explore the country-level association and longitudinal trends between the levels of WASH and typhoid fever burden.

## Conclusions

Our study findings will be useful to infer actionable insights on the most effective ways to control typhoid fever in LMICs. For instance, our findings reinforce the previous findings that, in addition to infrastructure improvements, behavioural changes such as washing hands with soap have a significant impact on the risk of contracting typhoid fever [9]. While major infrastructural improvements are crucial to reduce the burden of typhoid fever, they require resources that are difficult to commit to in LMICs. On the other hand, behaviour interventions may be feasible, affordable, and effective options to reduce disease risk in LMICs.

### Supplementary Information

**Additional file 1. **

## Data Availability

All data and materials are publicly available in this published article and its GitHub repository.
