# Supplementary material for: Associations of water, sanitation, and hygiene with typhoid fever in case–control studies: a systematic review and meta-analysis
Source: BMC Infect Dis. 2023 Aug 29;23:562. doi: 10.1186/s12879-023-08452-0 (PMC10464135; doi:10.1186/s12879-023-08452-0)
Supplement: Supplementary file 1 — Additional file 1. [file 12879_2023_8452_MOESM1_ESM.docx]

**Supplementary appendix**

**Appendix A. Data extracted from the included studies** This file contains information we extracted from the included papers. This was used as an input for the statistical analysis. (Author = author name; Year = publication year; Study = year and author, Country = country, Exposures = details of exposure, Measures = level of exposure; JMP WASH Category = classified category using JMP WASH category; Brockett Category = category used in the previous review; Crude OR = odds ratio from univariate analysis; Crude OR CI = confidence interval of odds ratio from univariate analysis; Adjusted OR = odds ratio from multivariate analysis; Adjusted OR CI = confidence interval of odds ratio from multivariate analysis; Diagnostic Methods = diagnostic methods used to define typhoid fever; Blood Culture = blood culture-based diagnosis of typhoid; Controlled Variable (All) = controlled variables in the meta-analysis; Controlled variables (other WASH factors) = other WASH exposures controlled)

[Download](https://spreadsheets.google.com/feeds/download/spreadsheets/Export?key=1ArKNfpa124oPdrbGK1-9PnzRWWvffsxgOUfgCVs_p2Q&exportFormat=csv) (download cvs file)

**Appendix B. Funnel plots** To assess publication bias, we evaluated funnel plot asymmetry. Significant funnel plot asymmetry was not detected in our analyses.


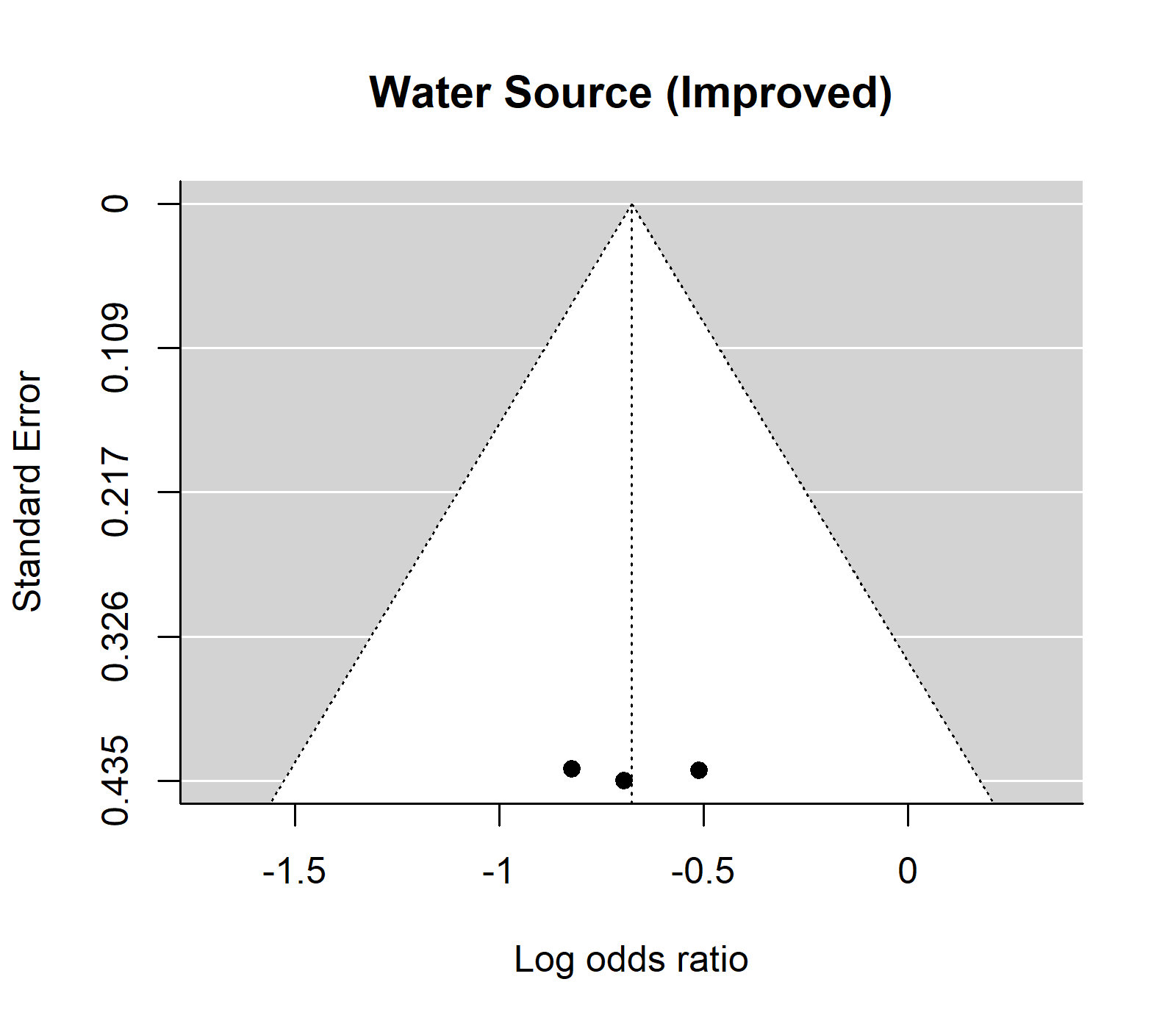

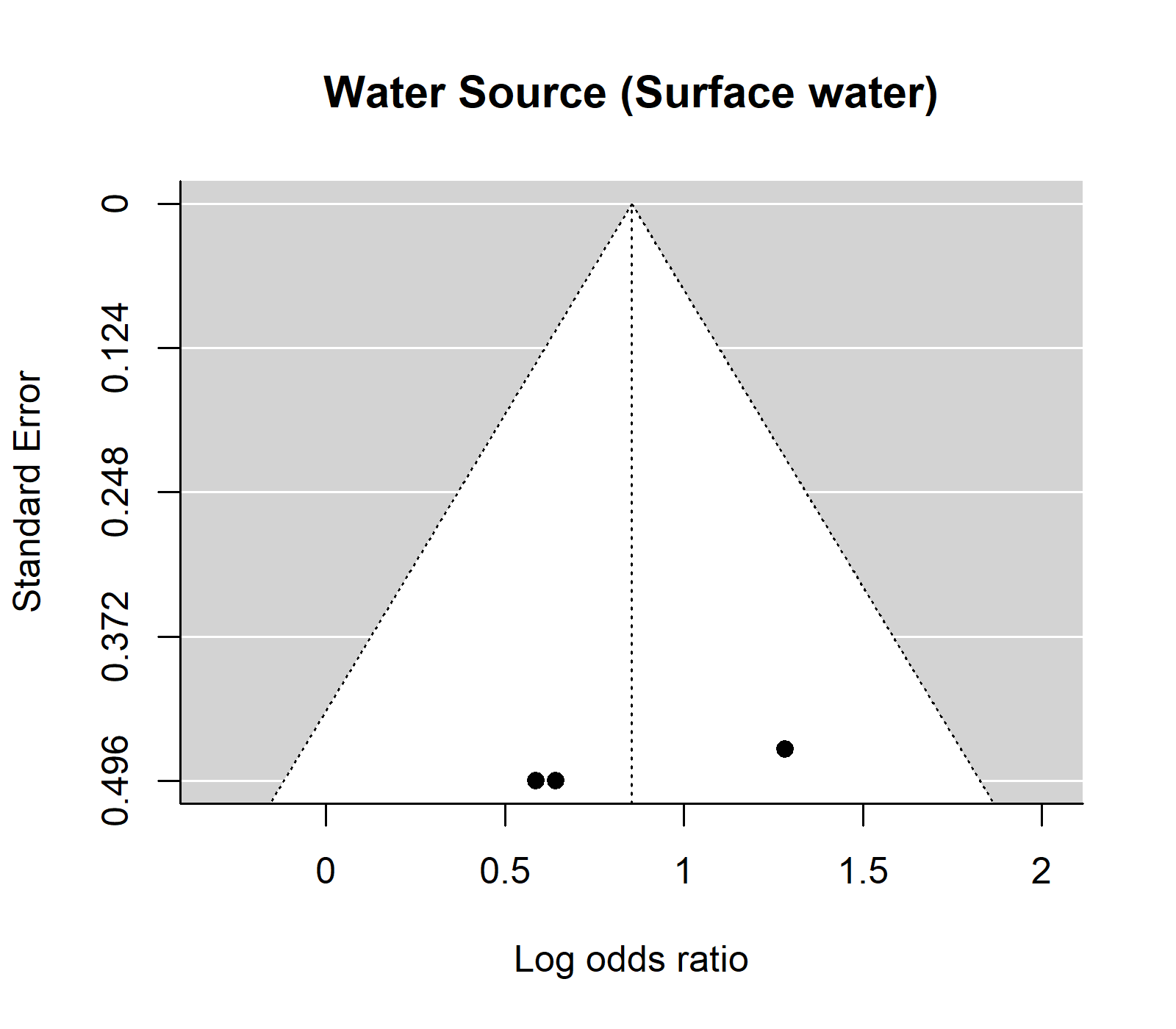


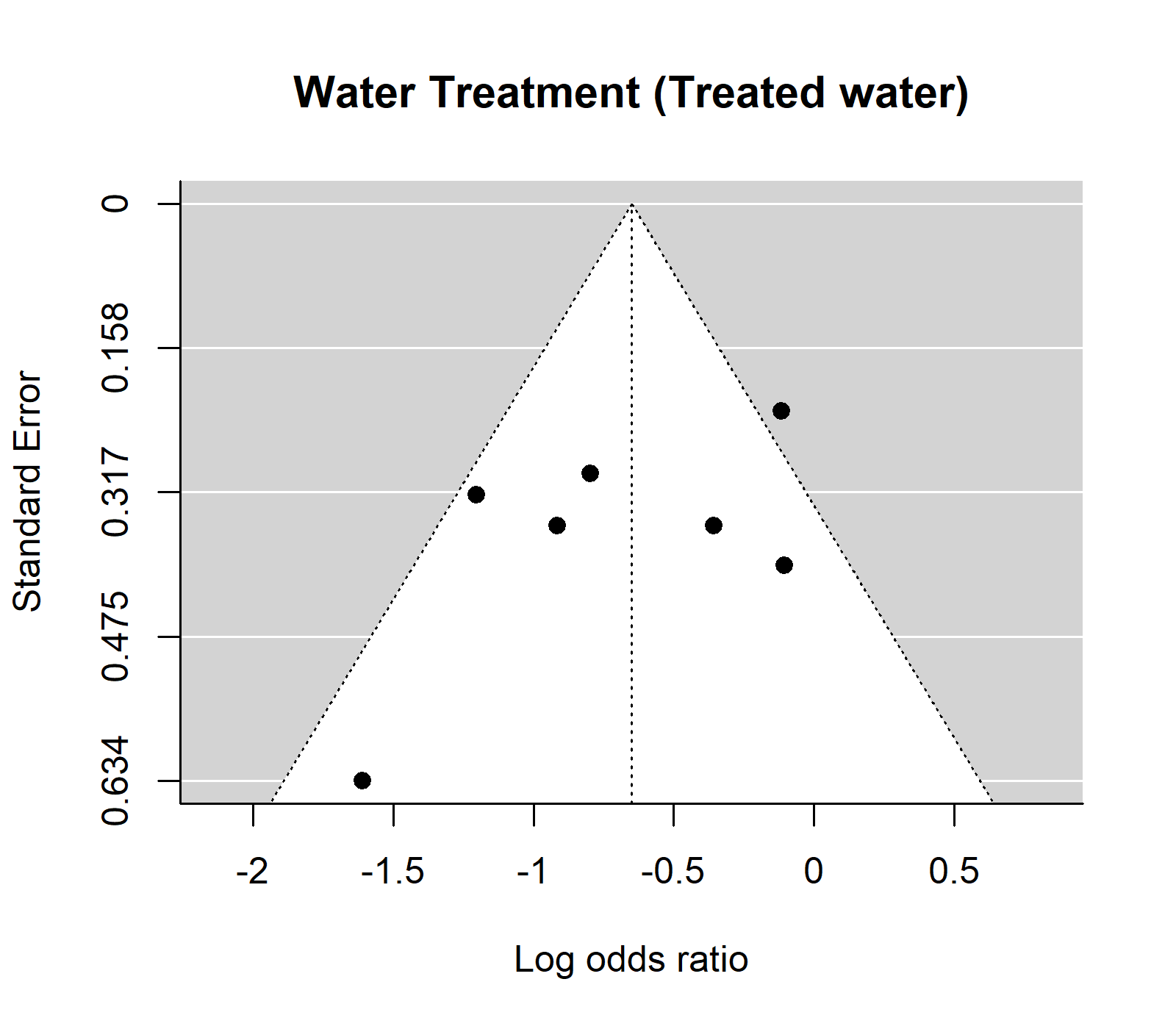

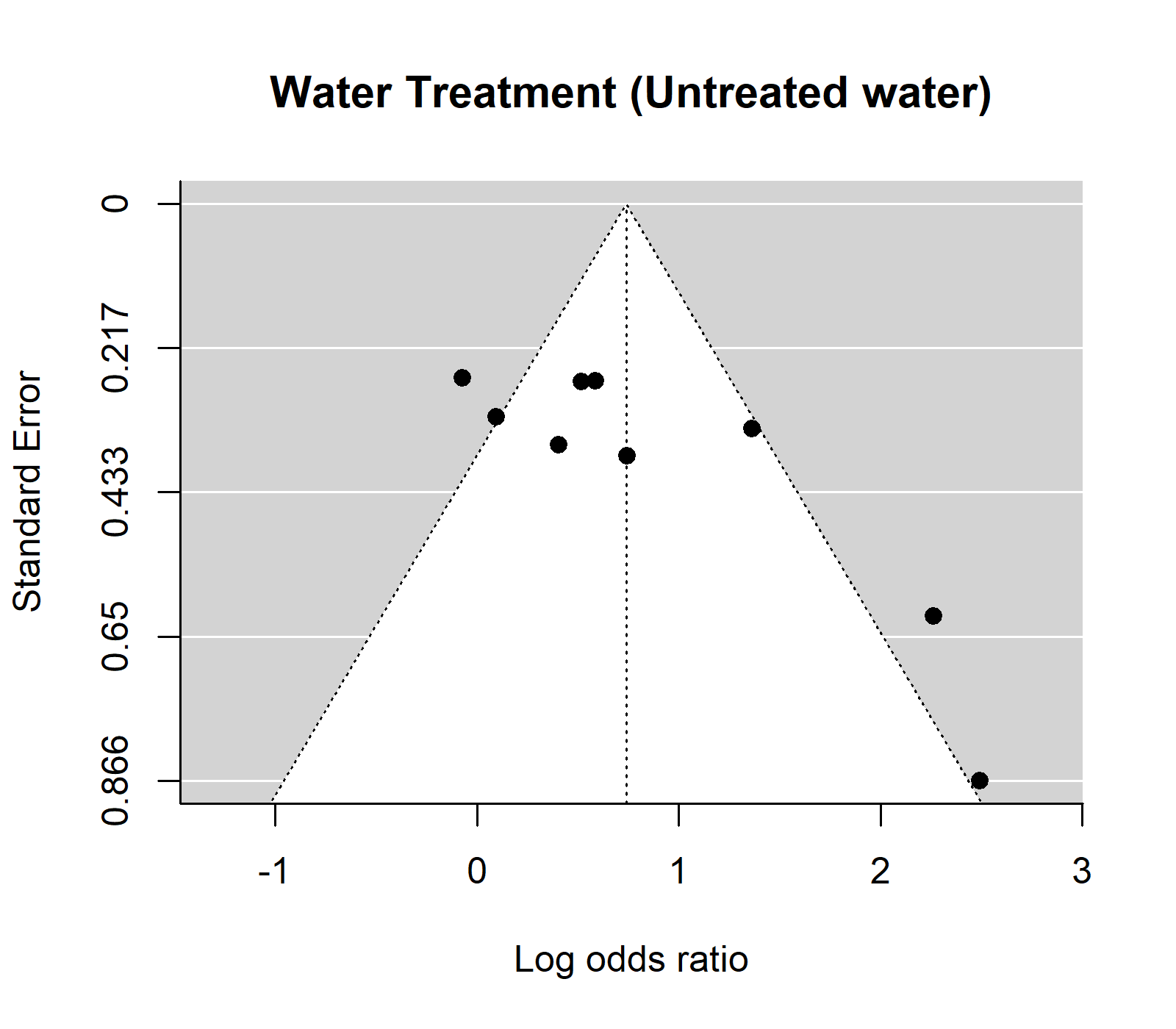


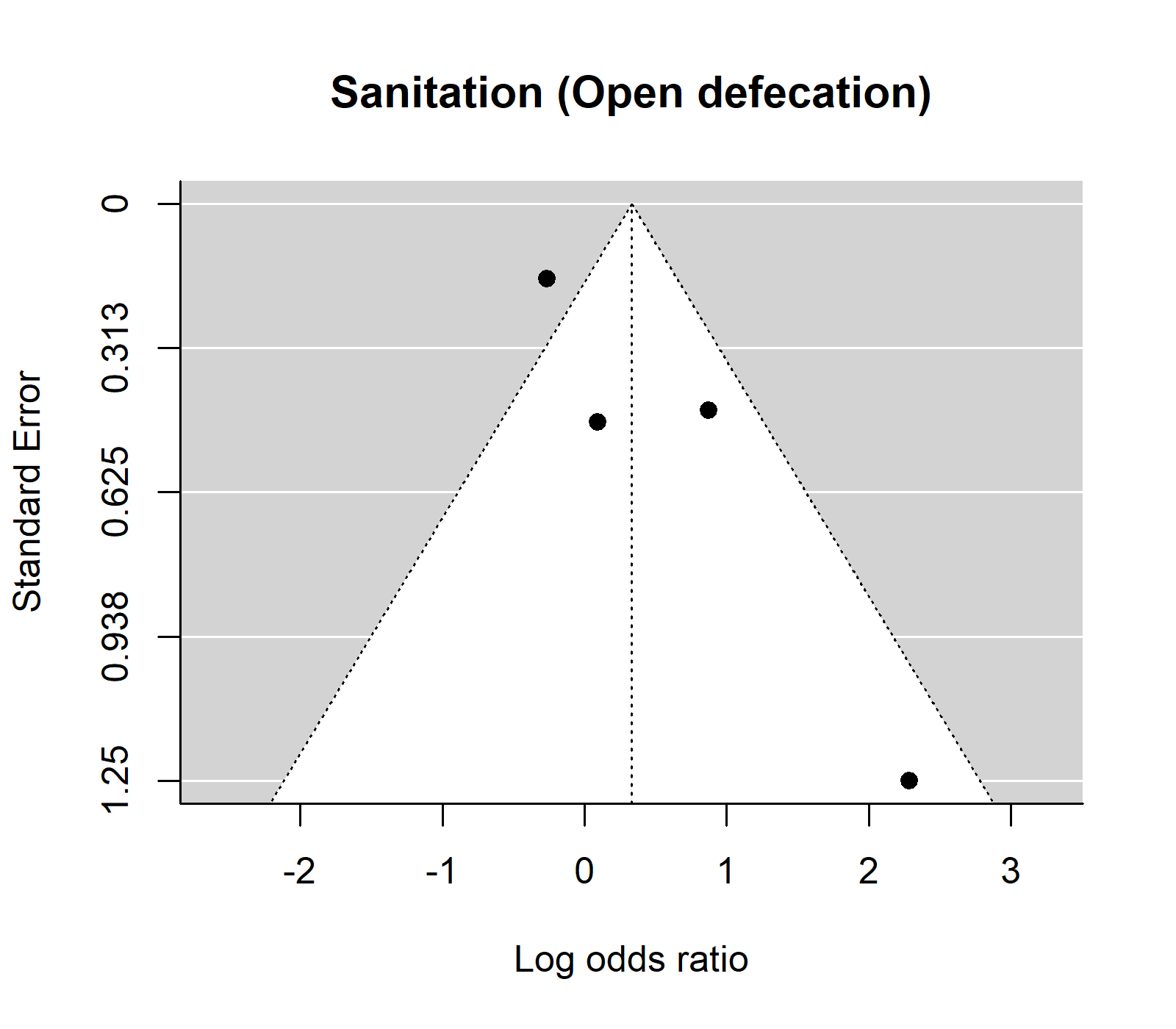


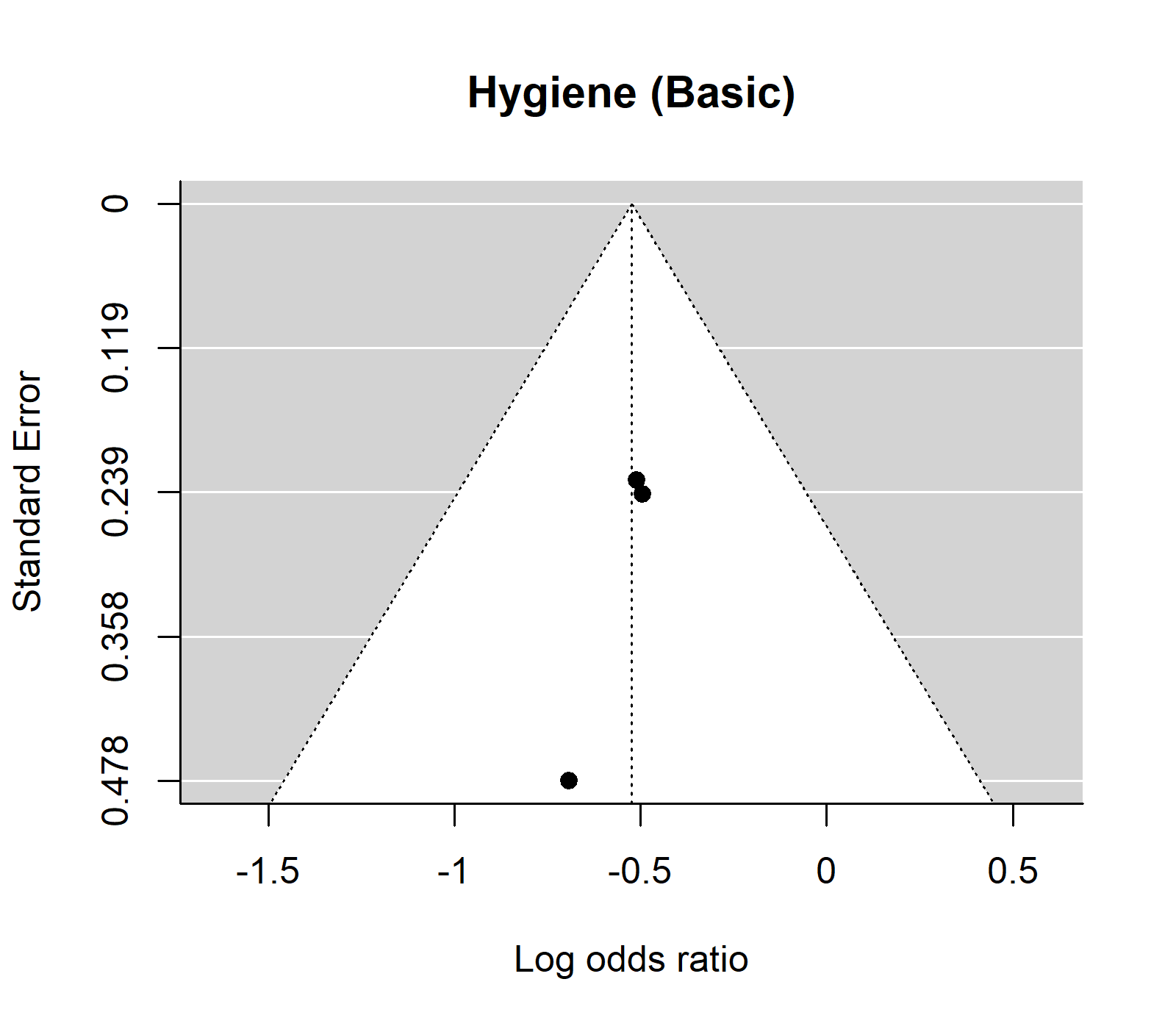

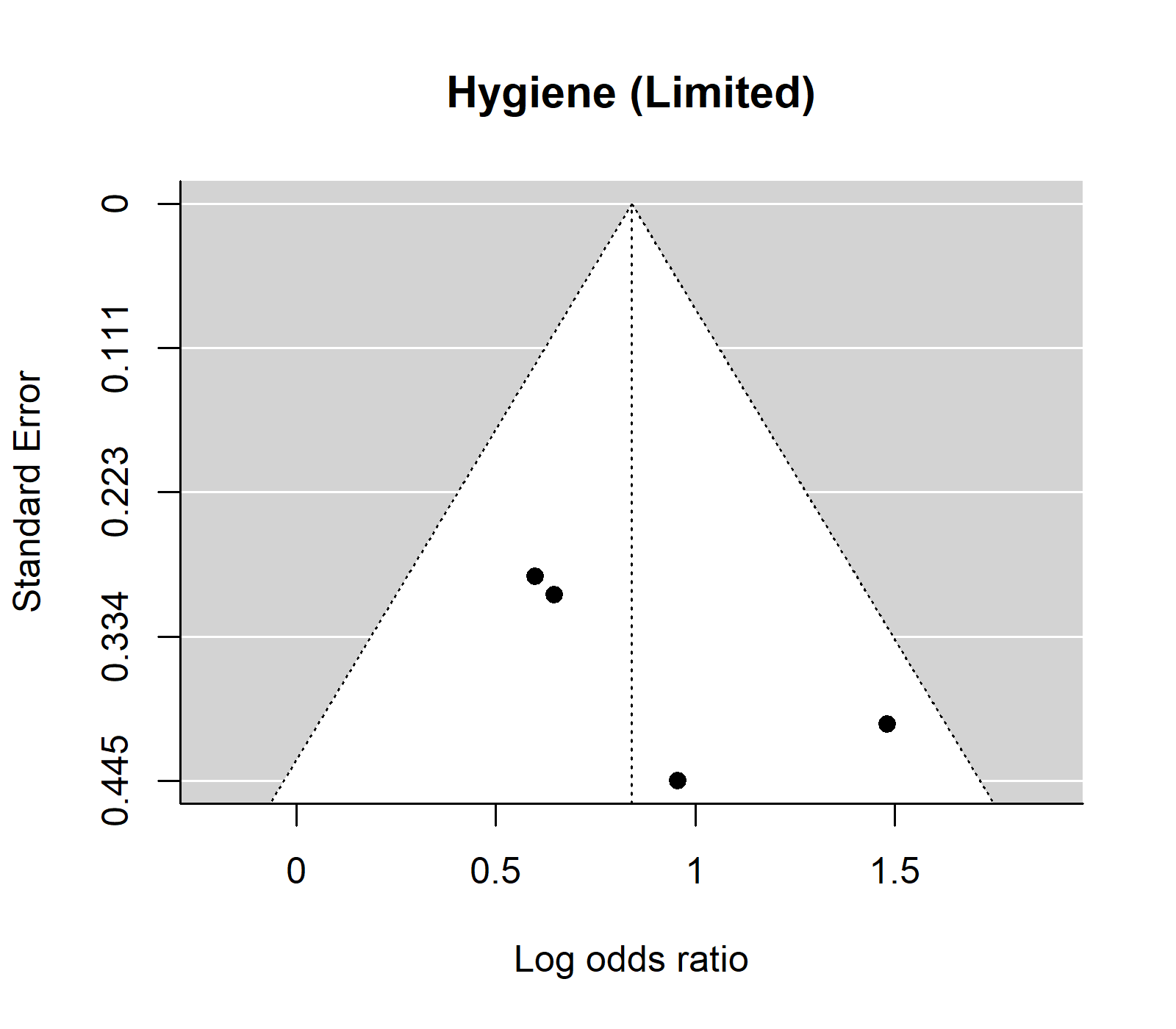


**Appendix C. Risk of bias assessment results** This is risk of bias assessment results broken down for each risk of bias criterion.


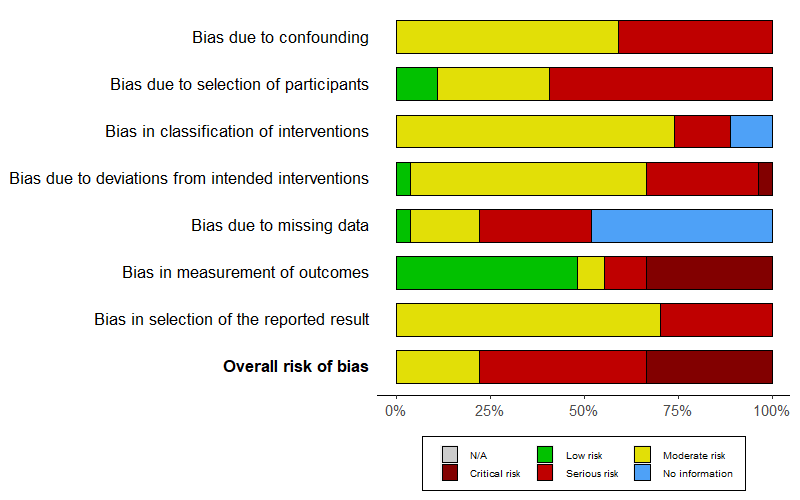


**Appendix D. Model description** This model describes the Bayesian random effects model used in our study

As below, observation, $y_{i}$ (i.e., the log(ORs) from the study $i$) is assumed to be normally distributed for a given mean $\theta_{i}$and standard error $\sigma_{i}$. Here, $\theta_{i}$is again assumed to be a normal random variable with a mean $\mu$ and variance $\tau^{2}$.

$y_{i}\sim$ Normal$(\theta_{i}, {\sigma_{i}}^{2})$

$\theta_{i}\sim$ Normal$(\mu, \tau^{2})$

$\mu\sim$ Normal$(0, 1)$

$\tau\sim$ Half-Cauchy$(0, 0.5)$

Our main interest is to estimate the true pooled effect size $\mu$ and the between-study heterogeneity$, \tau$. We defined prior distribution of μ as a normal distribution and τ as a half-Cauchy distribution with the location parameter of 0 and the scaling parameter of 0.5. The overall convergence and validity of the model were confirmed using the potential scale reduction factor and posterior predictive checks. Based on the assessments, we used 10,000 iterations of the MCMC algorithm.

**Appendix E. Association between potential protective/risk WASH factors and typhoid fever.** The forest plot illustrates the association between potential protective/risk WASH factors and typhoid fever. Filled circles are posterior median values. Thick and thin black lines show 80% and 95% credible intervals, respectively.

(A)


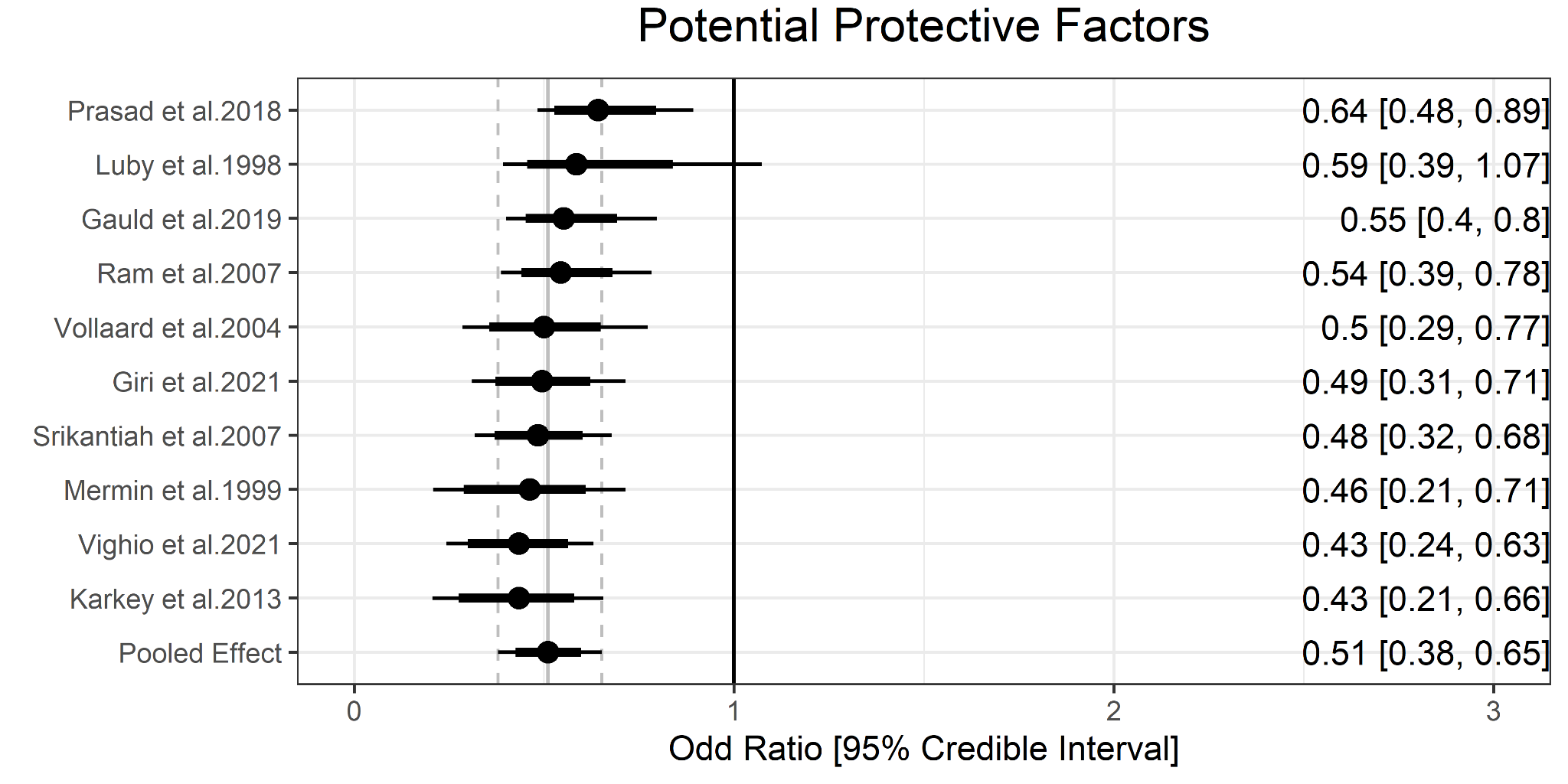


(B)


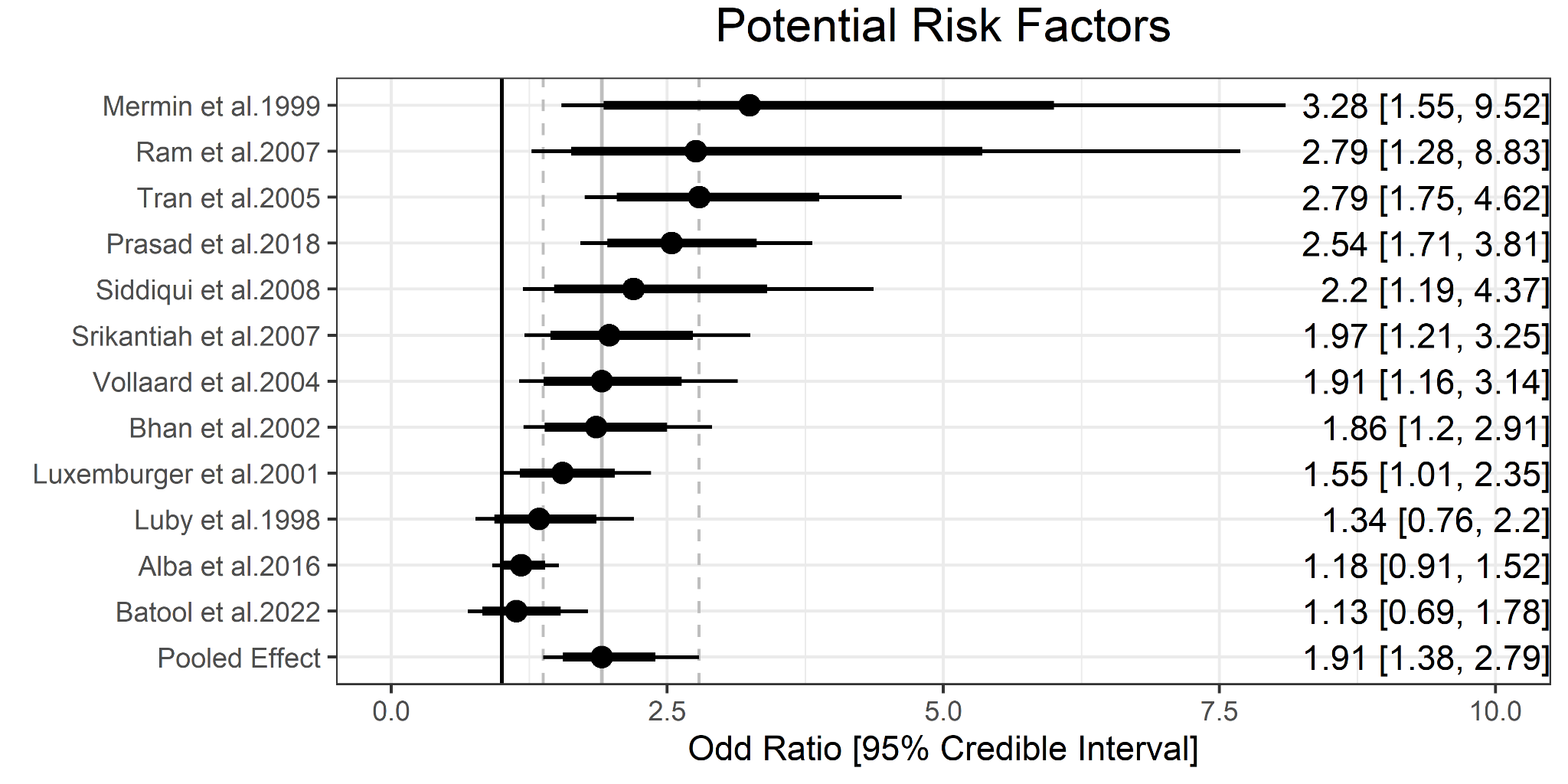


**Appendix F. Meta-analyses of WASH exposures and typhoid fever.** This table shows the pooled estimates using both frequentist and Bayesian approaches. The results of the Bayesian meta-analyses of WASH exposures and typhoid fever are shown with pooled odds ratio and 95% credible intervals. Standard deviation (SD) of true effects represents τ value. Heterogeneity in frequentist meta-analysis was assessed by using the $\boldsymbol{I}^{\boldsymbol{2}}$ statistic. The results were also compared with the previous studies of Brockett et al [(12)](https://sciwheel.com/work/citation?ids=12117344&pre=&suf=&sa=0) and Mogasale et al (6).

| **WASH indicators** | ***Current study*** | | | | | ***Previous studies*** | |
| --- | --- | --- | --- | --- | --- | --- | --- |
|  | ***N**** | **Pooled OR**  **[95% CrI]**† | **SD** | **Pooled OR**  **[95% CI]**†† | $\boldsymbol{I}^{\boldsymbol{2}}$ | **Brockett**  **(2020)** | **Mogasale**  **(2018)** |
| Improved water source | 3 | 0.54 [0.31, 1.08] | 0.29 | 0.51 [0.31, 0.83] | 0.00 % | 0.73 [0.56, 0.95] | 0.70 [0.46, 1.05] |
| Treated Water | 7 | 0.54 [0.36, 0.8] | 0.37 | 0.52 [0.36, 0.76] | 54.83% | 0.59 [0.45, 0.75] | NA |
| Basic hygiene | 3 | 0.60 [0.38, 0.97] | 0.24 | 0.59 [0.44, 0.81] | 0.00% | 0.52 [0.40, 0.67] | NA |
| Surface water | 3 | 2.14 [1.03, 4.06] | 0.35 | 2.35 [1.36, 4.08] | 0.00 % | 1.85 [1.37, 2.49] | NA |
| Untreated water | 9 | 1.96 [1.28, 3.27] | 0.55 | 2.09 [1.33, 3.29] | 73.97% | 2.39 [1.95, 2.93] | NA |
| Open defecation | 4 | 1.21 [0.64, 3.41] | 0.56 | 1.40 [0.66, 2.97] | 66.43% | 0.99 [0.84, 1.18] | NA |
| Limited hygiene | 4 | 2.26 [1.38, 3.64] | 0.29 | 2.32 [1.60, 3.36] | 15.95% | 2.20 [1.86, 2.60] | NA |

*Number of exposures

† Pooled estimates using Bayesian meta-analysis approach

†† Pooled estimates using frequentist meta-analysis approach

NA - not available
